# Solvothermal Synthesis of Rare Earth Bisphthalocyanines

**DOI:** 10.3390/molecules29112690

**Published:** 2024-06-06

**Authors:** Lina M. Bolivar-Pineda, Carlos U. Mendoza-Domínguez, Petra Rudolf, Elena V. Basiuk, Vladimir A. Basiuk

**Affiliations:** 1 Instituto de Ciencias Nucleares, Universidad Nacional Autónoma de México, Circuito Exterior C.U., Ciudad de México 04510, Mexico; 2 Zernike Institute for Advanced Materials, University of Groningen, Nijenborgh 4, 9747 AG Groningen, The Netherlands; 3 Instituto de Ciencias Aplicadas y Tecnología, Universidad Nacional Autónoma de México, Circuito Exterior C.U., Ciudad de México 04510, Mexico; elena.golovataya@icat.unam.mx

**Keywords:** synthesis, bisphthalocyanines, solvothermal method, sublimation

## Abstract

Rare earth bisphthalocyanines (MPc_2_) are of particular interest because of their behavior as single-molecular magnets, which makes them suitable for applications in molecular spintronics, high-density data storage and quantum computation. Nevertheless, MPc_2_ are not commercially available, and the synthesis routes are mainly focused on obtaining substituted phthalocyanines. Two preparation routes depend on the precursor: synthesis from phthalonitrile (PN) and the metalation of free or dilithium phthalocyanine (H_2_Pc and Li_2_Pc). In both options, byproducts such as free-base phthalocyanine and in the first route additional PN oligomers are generated, which influence the MPc_2_ yield. There are three preparation methods for these routes: heating, microwave radiation and reflux. In this research, solvothermal synthesis was applied as a new approach to prepare yttrium, lanthanum, gadolinium and terbium unsubstituted bisphthalocyanines using Li_2_Pc and the rare earth(III) acetylacetonates. Purification by sublimation gave high product yields compared to those reported, namely 68% for YPc_2_, 43% for LaPc_2_, 63% for GdPc_2_ and 62% for TbPc_2_, without any detectable presence of H_2_Pc. Characterization by infrared, Raman, ultraviolet–visible and X-ray photoelectron spectroscopy as well as elemental analysis revealed the main featuresof the four bisphthalocyanines, indicating the success of the synthesis of the complexes.

## 1. Introduction

Rare earth bisphthalocyanines, also known as double-decker phthalocyanines, exhibit a distinctive sandwich structure (MPc_2_ = where M refers to metals such as Y, Sc and lanthanide metal) are molecules where two phthalocyanine ligands coordinate to trivalent cations (M(III)). MPc_2_ has been reported to undergo ligand-centered redox-triggered interconversion between three forms of trivalent metal complexes—anionic [M^3+^(Pc^2−^)_2_]^−^, neutral radical [(Pc^−^)M^3+•^(Pc^2−^)]^0^ and cationic [M^3+^(Pc^−^)_2_]^+^ characterized by different colors: blue, green and red, respectively [1,2]. The flexibility of incorporating various substituents at the periphery and non-periphery of the macrocycle as well as at axial positions enables the fine-tuning of their physico-chemical properties, offering promising avenues for diverse applications. Furthermore, modifications can be made on the phthalocyanine ring, resulting in so-called Pcs analogs [3].

Bisphthalocyanines behave as single-molecule magnets and therefore attracted significant interest in magnetic storage and molecular spintronics associated with magnetic bistability applications [2,4,5,6,7]. Synthetic routes for preparing bisphthalocyanines both with and without substituents can be classified according to the type of precursors involved. Among the best known are tetramerization reactions of phthalic acid derivatives, mainly phthalonitrile, in the presence of the respective rare earth ions (also called template synthesis) and metal insertion reactions into preformed macrocyclic systems such as free-base phthalocyanine and/or lithium phthalocyanine [8,9,10,11].

Template synthesis, although straightforward, requires the precise control of the reaction conditions to achieve the desired selectivity (see review of the reference [8,9]) due to the formation of stable single-, double- and triple-decker complexes by rare earth ions. Reaction parameters decisive for the specific outcome are the temperature, time, type of rare earth salt precursor, type of phthalic acid derivate and ratio of reactants [8,9]. Three template synthesis methods have been reported, namely heating a mixture of rare earth acetates and phthalonitrile to a temperature of 250 to 350 °C (thermal fusion) [8,9], applying microwave radiation to provide the necessary energy for the reaction to occur and inducing a condensation reaction by the reflux of the above mixture in the presence of DBU (1,8-Diazabicyclo[5.4.0]undec-7-ene) as a strong organic base and alcohol with a long chain (pentanol and hexanol) [8,9]. The drawback of this synthesis route is the formation of oligomers from the precursor used, e.g., from PN and also from H_2_Pc [8,9].

Double-decker phthalocyanines can also be prepared by reflux and microwave irradiation starting from free-base and lithium phthalocyanines with rare earth acetates or acetylacetonates (M(acac)_3_) in quinoline, trichlorobenzene and/or *n*-octanol (basic medium). Unlike using phthalic acid derivatives such as PN as a precursor, using H_2_Pc and Li_2_Pc only produces H_2_Pc as a byproduct [8,9]. Purification techniques such as column chromatography and sublimation are frequently employed, the former being particularly common.

The synthesis routes and preparation methodologies mentioned above have mainly focused on obtaining bisphthalocyanines with substituents on their aromatic rings, using chromatography as the purification technique. It is well known that unsubstituted phthalocyanines have low solubility in conventional low-boiling solvents (<100°), which limits their applications. Therefore, a large number of studies have focused on substituted bisphthalocyanines which are more soluble. Despite this, the latter are less thermally stable, as this depends on the nature of the substituent [3,8,9,10,11].

As for the percentage yields for substituted phthalocyanines using precursors such as PN, they vary between 2% and 80%, this being the most widely used method. With H_2_Pc and Li_2_Pc, the yields range from approximately 9% to 90%. In comparison, the yields for the unsubstituted are lower, ranging from 5% to 60% for PN and 9% to 33% for H_2_Pc and Li_2_Pc [8,9].

**Scheme 1:**
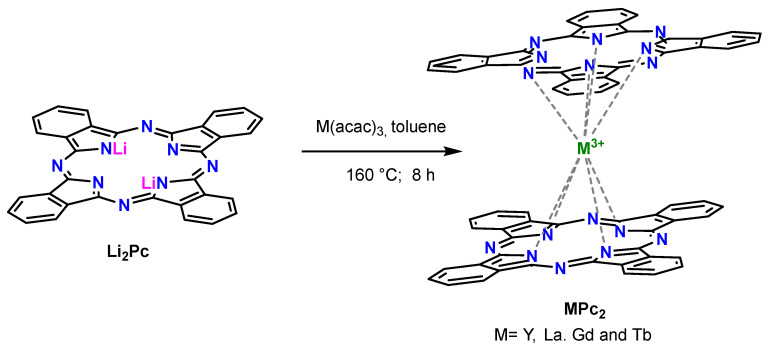
Synthesis route of rare earth bisphthalocyanines (MPc_2_ = YPc_2_, LaPc_2_, TbPc_2_ and GdPc_2_).

The selection of a suitable method for the preparation of phthalocyanines is important for their properties and the cost of production. Herein, we propose a novel synthesis strategy for rare earth bisphthalocyanines—yttrium, lanthanum, gadolinium and terbium—utilizing the solvothermal method. This approach involves the use of acetylacetonates and dilithium phthalocyanine precursors in toluene (Scheme 1), providing a promising alternative to existing methods. The solvothermal method offers operational simplicity, environmental friendliness and direct transformation from reactants to products, leading to crystalline structures with controllable morphologies [12,13]. Despite the versatility and effectiveness of the solvothermal method in synthesizing single-decker phthalocyanines [12,13,14,15,16], rare earth bisphthalocyanines have remained unexplored using this approach. Hence, this work aims to fill this gap by synthesizing rare earth bisphthalocyanines via the solvothermal method, paving the way for tailored applications in various fields.

## 2. Results and Discussion

The synthesis of rare earth double-decker phthalocyanines, such as yttrium, lanthanum, gadolinium and terbium using the metalation of lithium phthalocyanines, offers the advantage of avoiding the formation of some unwanted byproducts, such as linear oligomers of phthalonitrile [8]. However, the presence of free-base phthalocyanine as a contaminant remains unavoidable [17]. To address this, the crude product was purified by sublimation at a constant temperature of 350 °C for two or three hours, depending on the central metal of the MPc_2_. Table 1 shows the yields obtained for each of the bisphthalocyanines: 68% for YPc_2_, 43% for LaPc_2_, 63% for GdPc_2_ and 62% for TbPc_2_; these percentages were calculated based on dilithium phthalocyanine, the limiting reactant.

The parameters to compare the percentage yield with the data reported in the literature are the synthesis route and purification method. The yield of the solvothermal method used to synthesize unsubstituted bisphthalocyanines is higher than when the insertion of the rare earth metal into Li₂Pc with acetylacetonates is conducted by refluxing and/or microwave irradiation and whose purification is performed by column chromatography (12–33%) [8].

The purification method plays a crucial role in the percentage yield. The purification of five unsubstituted bisphthalocyanines by sublimation under nitrogen atmosphere in 5–60% yield was reported [17]. Rare earth acetate and phthalonitrile were used as precursors under simultaneous heating and stirring [17]. These results underline the efficiency of our solvothermal and sublimation method.

The synthesis route of the four bisphthalocyanines in this work was chosen because the use of Li_2_Pc produces only H_2_Pc as a byproduct, which allowed for H_2_Pc to be removed from the product of interest by sublimation. H_2_Pc molecules have the ability to sublimate at high temperatures without decomposing [10]. The presence of H_2_Pc was qualitatively detected in the quartz tube at the end of the sublimation process. Table 1 displays the content of H_2_Pc in the washed end product.

**Figure 1:**
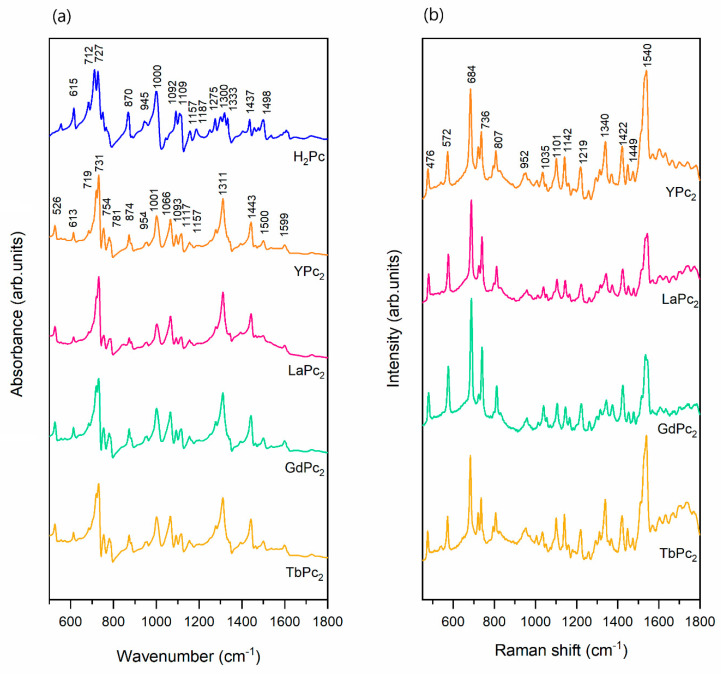
(**a**) FTIR and (**b**) Raman spectra of free-base and of four rare earth double-decker phthalocyanines: YPc_2_, LaPc_2_, GdPc_2_ and TbPc_2_.

**Figure 2:**
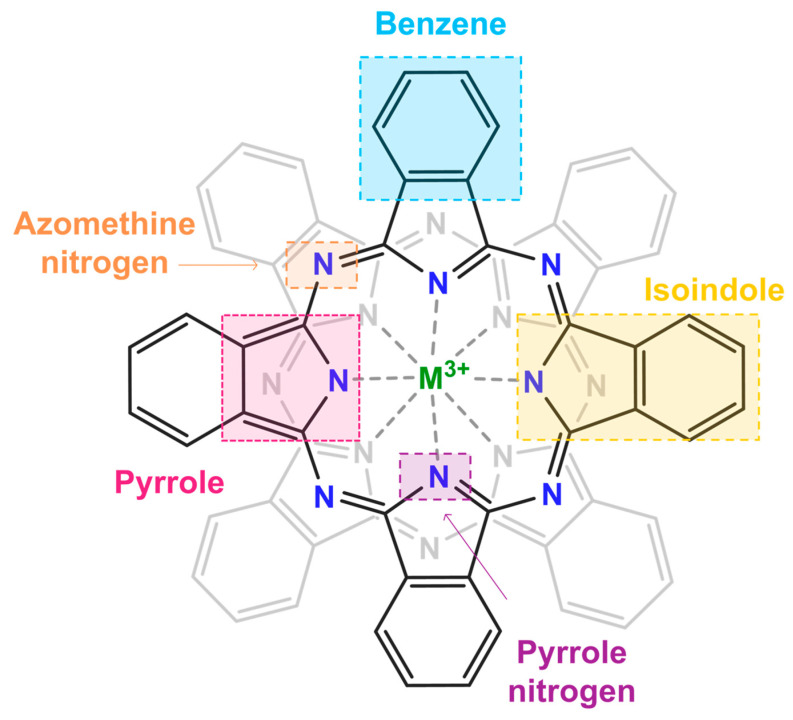
Typical chemical structure of rare earth double-decker phthalocyanines. M^3+^ = Y, La, Gd and Tb.

Fourier-transform infrared spectroscopy (FTIR) was employed to detect the characteristic features of the unsubstituted MPc_2_ [17,18,19]. As depicted in Figure 1a, the spectra of the four rare earth double-decker phthalocyanines exhibit the characteristic vibrational modes of phthalocyanines ligands in the region of 500–1800 cm^−1^ reported in the literature [17,18,20,21,22,23,24,25,26,27]. Notably, five main IR absorptions with strong and medium intensity were observed in all four spectra. For a comprehensive understanding of the vibrational modes present in the infrared (Figure 1a) and Raman spectra (Figure 1b). Figure 2 depicts the detailed structure of the MPc_2_.

The highest intensity band at 731 cm^−1^ is attributed to C-H out-of-plane bending, while the absorption at 1001 cm^−1^ is assigned to the pyrrole N in-plane bending of the phthalocyanine ligand. The band at 1066 cm^−1^ is due to the coupling of the isoindole deformation and the aza group stretching (C=N), and the ones at 1311 and 1443 cm^−1^ arise from pyrrole and isoindole stretching [20,21,27]. Another band typical of these complexes is the weak intensity feature at 1117 cm^−1^ that corresponds to an isoindole breathing mode with small contributions from C-H in-plane bending [20,21].

The occurrence of a pyrrole stretching band at 1311 cm^−1^ implies the presence of a phthalocyanine monoanion radical, Pc^•−^, suggesting the existence of one unpaired electron in one of the tetrapyrrole rings [18,20,21]. The absence of a band around 1329 cm^−1^ (Figure 1a) supports the delocalization of the unpaired electron over both phthalocyanine rings; that is, there are no dianionic phthalocyanine ligands (${\left[{MPc}_{2}\right]}^{-}$) [26]. The weak bands at 874 and 1500 cm^−1^ derive from isoindole, pyrrole deformation and the aza stretching mode. The other weak bands that appear between 500 and 1000 cm^−1^ represent Pc ligand breathing and C-H out-of-plane vibrations, whereas the bands in the range from 1000 to 1300 cm^−1^ are due to C-H in-plane modes [20,21]. The vibrations in the region from 1344 to 1479 cm^−1^ and from 1537 to 1599 cm^−1^ are attributed to isoindole and benzene stretching modes, respectively [20,21], while the set of bands at 3022–3271 cm^−1^ is due to aromatic C-H stretching in the phthalocyanine rings (for further detail, see Table S1 and Figure S1 of Supporting Information) [20,21].

Comparing the infrared spectrum of each of the bisphthalocyanines with the infrared spectrum of free-base phthalocyanine, it appears that the presence of the latter as an impurity is minimal. It can be seen that in the region of 1000 to 1600 cm^−1^ in the spectrum of the bisphthalocyanines, the band 1311 cm^−1^ stands out, which is a typical band for neutral rare-earth bisphthalocyanines [18,20,21]. Slight discrepancies between the FITR spectra (Figure 1a) and spectra in the literature are attributed to variations in sample preparation and to the fact that most of the reported spectra were obtained from MPc_2_ in thin films [22,24] and KBr pellets [19,20,23,24], while here, powder samples were analyzed, which implies differences in molecular orientation and the direction of the incident light [24]. Despite this, the spectra in Figure 1a demonstrate that the synthesized compounds were consistent with the expected target products.

As can be expected, the Raman spectra at room temperature show characteristic Raman shifts for typical rare earth double-decker phthalocyanines (Figure 1b and Table S2). In detail, the bands in the range of 476–807 cm^−1^ are assigned to phthalocyanine breathing, except for the band at 736–740 cm^−1^, which is due to aromatic phthalocyanine C-H wagging. Bands with different intensity appearing in the range of 900 to 1300 cm^−1^ are attributed to aromatic C-H bending. The bands at around 1141–1145 cm^−1^ and 1536–1544 cm^−1^ are due to pyrrole breathing modes, while those in the range of 1350–1600 cm^−1^ originate from isoindole ring stretching vibrations and from the aza group stretching, and the lines between 1420–1425 cm^−1^ and 1448–1453 cm^−1^ are typical of isoindole stretching [20,21,28,29,30]. The conclusion that YPc_2_, LaPc_2_, GdPc_2_ and TbPc_2_ were successfully synthesized is also supported by the elemental analysis of each product (Table 1), which aligns well with the mass percentages of the four rare earth bisphthalocyanines and by the X-ray photoelectron spectroscopy (XPS) spectra of the bisphthalocyanines.

**Figure 3:**
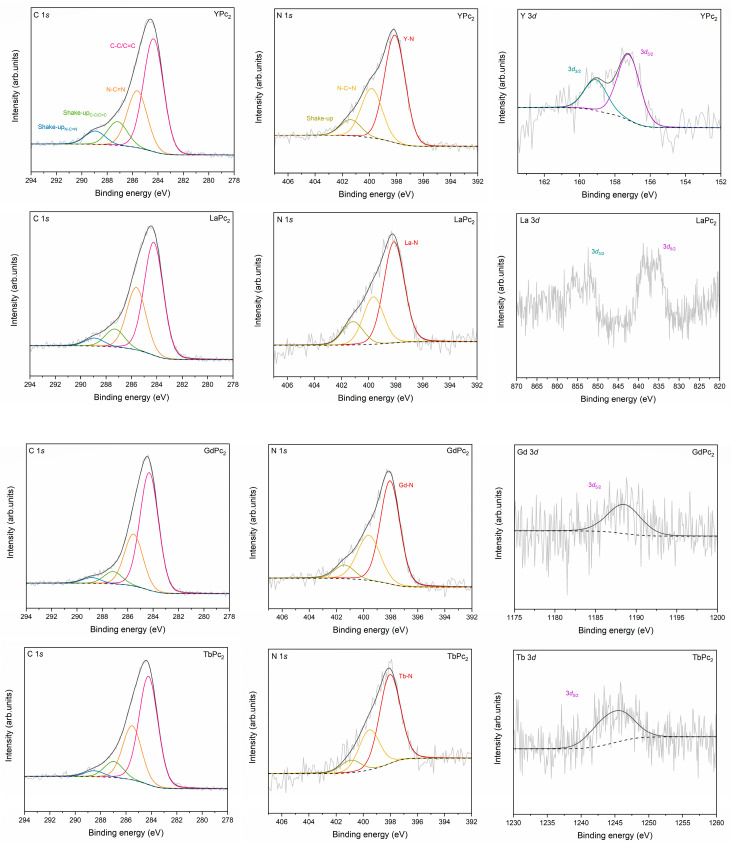
The XPS spectra of the C 1*s*, N 1*s* and metal 3*d* core-level region of yttrium, lanthanum, gadolinium, and terbium bisphthalocyanines.

In XPS, not only can the elements present be identified but so can the chemical environment in which they are. Figure 3 shows the C 1*s*, N 1*s* and the 3*d* core-level regions of the respective rare earth metal for all four bisphthalocyanines. The C 1*s* spectra in the left panels of Figure 3 show four contributions; the most intense peak located at a binding energy (B.E.) between 284.5 and 284.7 eV corresponds to the 48 carbon atoms in the *sp*^2^ bonds of the benzene ring, and next to it, at a B.E between 285.6 and 286.9 eV is the contribution from carbon–nitrogen bonds, arising from the 16 pyrrole carbon atoms in the structure (N-C=N). At a higher B.E., one observes the shake-up satellites, namely the shake-up_C=C_ at a B.E. between 287.2 and 287.5 eV and the shake-up_N-C=N_ at around from 289.0 to 289.4 (Table S3) [31,32].

In N 1*s* core-level spectra, in the central panels of Figure 3, three contributions can be discerned, the one due to the nitrogen atoms of the isoindole units coordinated to the metal ion (M-N*_iso_*, where M = Y, La, Gd and Tb and N*_iso_* = nitrogen atom of isoindole or unit) at a BE between 398.0 and 398.2 eV, the peak stemming from azomethine nitrogen (γ-N) bonded with carbon atoms C-N=C at a BE from 399.5.0 to 400.0 eV and the shake-up features around 401.0 and 401.6 eV [33,34,35,36]. Table S4 reports the binding energies of the different components identified in the fits.

The confirmation of the formation of the coordination bond (M-N) in rare earth metal bisphthalocyanines comes from the M 3*d* core-level regions in the right panels of Figure 3; the binding energy values are reported in Table S4. For yttrium and lanthanum bisphthalocyanines, the orbital split 3 *d*_3/2_ and 3 *d*_5/2_ lines were observed, while for gadolinium and terbium bisphthalocyanines, only the 3 *d*_5/2_ core region could be identified, while the 3*d*_3/2_ was hidden in the noise. Unfortunately, the amount of the rare earth metal is so small that the low signal intensity does not allow us to extract more information.

**Figure 4:**
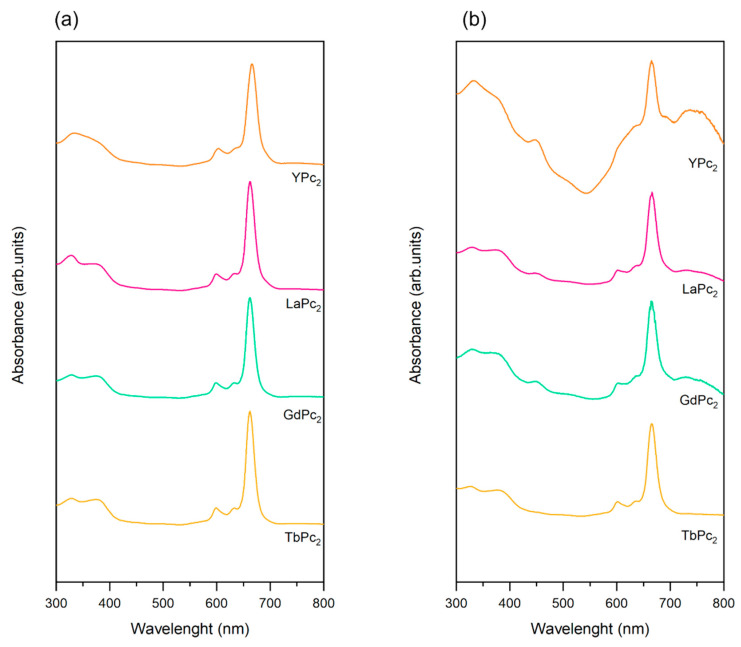
UV–visible spectra of four rare earth bisphthalocyanines: YPc_2_, LaPc_2_, GdPc_2_ and TbPc_2_ in (**a**) DMF and (**b**) DMSO.

The ultraviolet–visible (UV-vis) absorption spectra of the products obtained after the sublimation process were recorded in dimethylformamide (DMF) and dimethyl sulfoxide (DMSO) and are presented in Figure 4. It is important to mention that the electronic absorption of phthalocyanines depends on the chemical environment such as the type of solvent and its interaction with rare earth metal complexes. Hence, some discrepancies can be found between the UV-vis spectra of MPc_2_ complexes in thin films, solution and solid pellets [22,23,24,26,27,37,38,39,40,41,42,43]. The spectra in Figure 4 exhibit the two main characteristic bands of phthalocyanines, the Q and the B or Soret bands [26,39,40]. The Q band resulting from the π(a_1u_)-π*(e_g_) HOMO-LUMO transition that originates from electronic charge transfer from the pyrrole skeleton to the condensed benzene rings of Pc ligands [26,39] dominates the absorption and was observed between 662 nm (LaPc_2_. GdPc_2_ and TbPc_2_ in DMF) and 667 nm (GdPc_2_ in DMSO) in both solvents (see Table S3). Differences in absorption wavelengths are negligible between polar aprotic solvents, and the small hypochromic shift of the Q-band is due to the decrease in the ionic radii of the rare earth cations in the case of dimethylformamide solvent [26,40].

The Q-band of phthalocyanines in DMF presents a small peak near 599 nm for LaPc_2_, GdPc_2_ and TbPc_2_ while for YPc_2_, at 603 nm. In dimethyl sulfoxide, this band is only observed for LaPc_2_, GdPc_2_ and TbPc_2_ at 601–602 nm. It has been reported [26,40] that such a Q-band splitting indicates that these compounds have a strong tendency to aggregate. The B band that corresponds to an additional π (a_2u_)-π*(e_g_) transition is produced by the redistribution of the electronic density that provokes an increase in electron density in correspondence with the bridging atoms of the azamethine group [26,39]; it is localized between 333 nm (YPc_2_ in DMF and DMSO) and 329 nm for (GdPc_2_ in DMF) in DMF and DMSO (Table S5).

**Figure 5:**
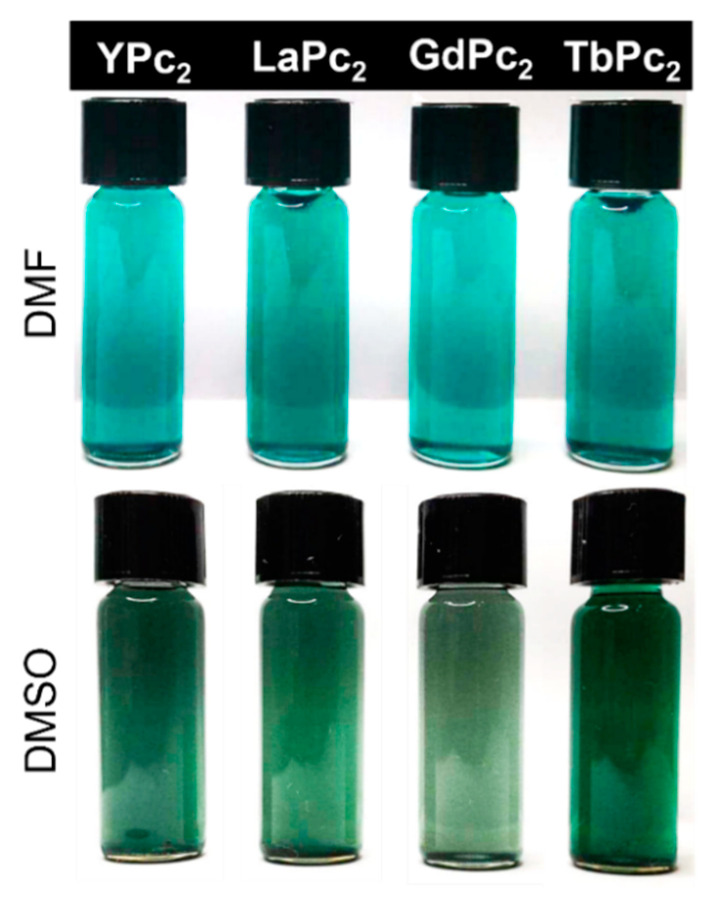
Photograph of solutions of four rare earth bisphthalocyanines: YPc_2_, LaPc_2_, GdPc_2_ and TbPc_2_ in DMF and DMSO.

An additional feature in the UV-vis spectrum of the double-decker phthalocyanines is the band around 450 nm, which appears as a result of electron delocalization between two aromatic systems in the neutral radical form of the metal complex [38,39,41]. This band can be identified in the spectra of yttrium, gadolinium and terbium bisphthalocyanines in dimethyl sulfoxide solutions (Figure 4b and Table S5). The absence of this band in the spectra obtained from the dimethylformamide solutions can be attributed to a possible reduction of the [MPc_2_]^•−^ complex to ${\left[{MPc}_{2}\right]}^{-}$ by the amine impurities that are often present in reagent-grade DMF [26]. This would explain the color difference between the DMSO and DMF solutions of the four double-decker phthalocyanines, as in the photograph in Figure 5. The blue-green color of the DMF solutions resulted from a mixture of the anionic and neutral form of bisphthalocyanines, while the dark and light green color of the dimethyl sulfoxide solutions is typical of the neutral bisphthalocyanines [1,26,39,43].

**Figure 6:**
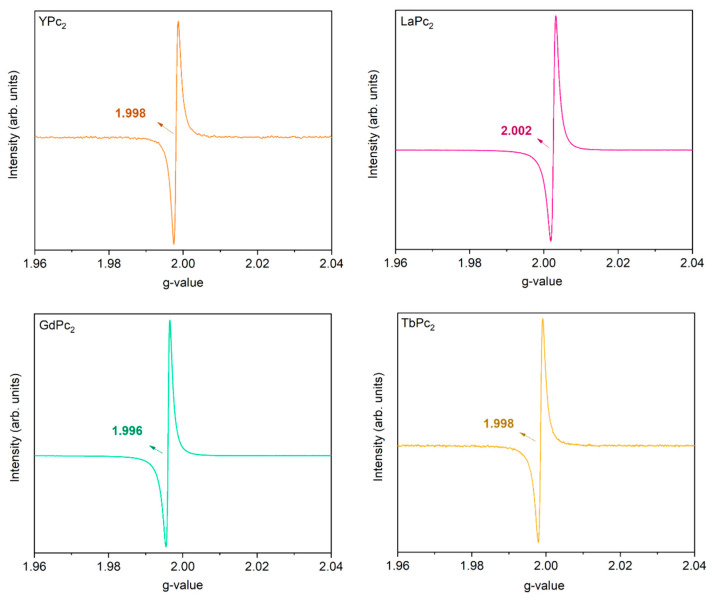
EPR spectra of four rare earth bisphthalocyanines: YPc_2_, LaPc_2_, GdPc_2_ and TbPc_2_ at room temperature.

The presence of an unpaired electron (free radical) in one of the Pc ligands of the bisphthalocyanines deduced from the FTIR spectra is confirmed by electron paramagnetic resonance (EPR, Figure 6). The spectra of all the complexes studied here in powder form show a strong and sharp isotropic Landé signal around a *g*-factor of 2.0 with a peak-to-peak linewidth of 0.2 mT, which is attributed to the 1/2 spin radical. This result is additional proof of the successful synthesis and underscores the paramagnetic nature of these rare earth sandwich structures [22,27,44].

**Figure 7:**
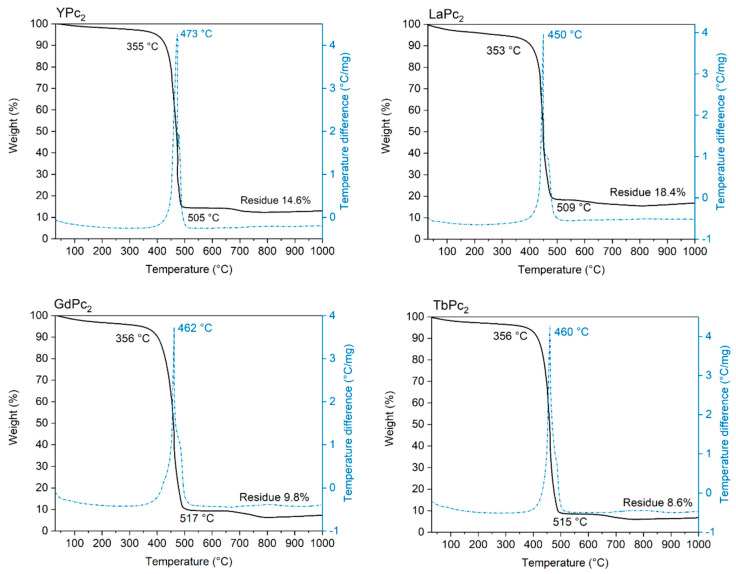
Thermogravimetric analysis (black) and differential thermal analysis (blue) of four rare earth double-decker phthalocyanines: YPc_2_, LaPc_2_, GdPc_2_ and TbPc_2_. Air flow during measurements was 100 mLmin^−1^.

The thermal stability of the four bisphthalocyanines was determined by thermogravimetric analysis (TGA) and differential thermal analysis (DTA). The corresponding curves presented in Figure 7 show a good agreement between TGA/DTA and reveal that all the MPc_2_ behave similarly upon heating. The first weight loss around 100–150 °C is attributed to moisture (a small amount of water absorbed upon storage in air). The main weight loss, which corresponds to the gradual oxidative pyrolysis of the double-decker complexes, comprises two main exothermic events. The first decomposition event occurs at 355 °C for YPc_2_, 353 °C for LaPc_2_, 359 °C for GdPc_2_ and 356 °C for TbPc_2_. The second event coincides with the maximum temperature of the DTA curves, and the final temperature is between 505 and 517 °C, illustrating that it is the strong conjugation within the macrocyclic ligand itself that governs the stability of phthalocyanine compounds, as already observed [45] for transition metal phthalocyanines and H_2_Pc. 

**Figure 8:**
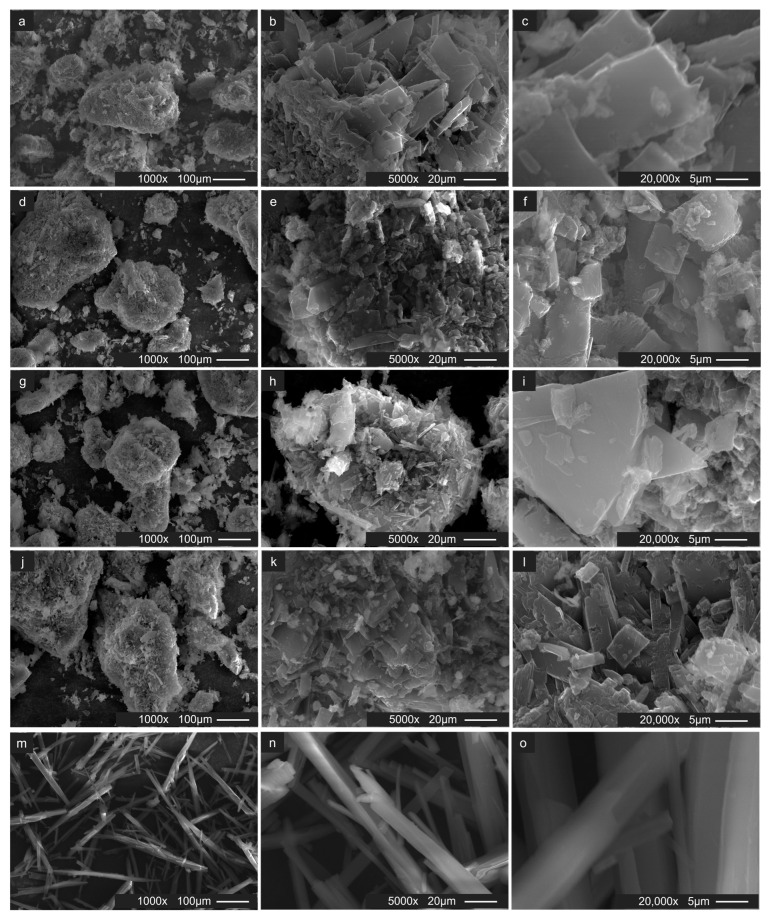
Representative SEM images at different magnifications for purified crystalline four rare earth double-decker phthalocyanines: (**a**–**c**) YPc_2_, (**d**–**f**) LaPc_2_, (**g**–**i**) GdPc_2_ (**j**–**l**) and TbPc_2_ and (**m**–**o**) crystalline commercial free-base phthalocyanine for comparison. Scale bars: (**a**,**d**,**g**,**j**,**m**) 100 μm, (**b**,**e**,**h**,**k**,**n**) 20 μm and (**c**,**f**,**i**,**l**,**o**) 5 μm.

Finally, scanning electron microscopy (SEM) was employed to explore the morphology of the synthesized bisphthalocyanine powders. Figure 8 exhibits scanning electron microscopy (SEM) images at different magnifications (Figure 8a–c for YPc_2_, Figure 8d–f for LaPc_2_, Figure 8g–i for GdPc_2_ and Figure 8j–l for TbPc_2_), revealing agglomerated and stacked crystallites with a laminar microstructure in the form of sheets with rectangular and/or square geometry (Figure 8e,f,i,l). In contrast, the SEM images of commercial free-base phthalocyanine H_2_Pc (Figure 8m–o) show needle-shaped crystals.

## 3. Materials and Methods

### 3.1. Materials

Dilithium phthalocyanine (70% purity) and the rare earth(III) acetylacetonate hydrates M(C_5_H_7_O_2_)_3_·xH_2_O (M = Y, La, Gd and Tb) of yttrium, lanthanum, gadolinium and terbium (all 99.9% purity) were acquired from Sigma-Aldrich (Burlington, MA, USA) and used as received.

### 3.2. Synthesis

All rare earth double-decker phthalocyanines were synthesized by the solvothermal method. Dilithium phthalocyanine (200 mg) and rare earth acetylacetates (600 mg) were used as precursors. For each type of rare earth acetylacetate, the precursors were mixed in a molar ratio of 1:3 in 60 mL of toluene in a stainless steel reactor with a polytetrafluoroethylene (PTFE—Teflon) and brought to a temperature of 160 °C in an oven for a time of 8 h. The reactor was then allowed to cool down to room temperature. The color of the solution after the reaction turned dark green, but as the solvent evaporated at room temperature, the solid began to precipitate, and the solution turned light green. The solid crude product had a dark blue-green-to-green coloration, depending on the metal used. The crude product was washed successively with methanol to eliminate the acetylacetates that had not reacted; every washing included an ultrasonication treatment (for about two minutes). The product acquired a color between dark green and purple, which points to the presence of free-base phthalocyanines H_2_Pc as byproducts. Hence, the purification of the different rare earth bisphthalocyanines was carried out in a train sublimation system under nitrogen atmosphere at 350 °C during two and three hours to remove free-base phthalocyanine.

### 3.3. Characterization

#### 3.3.1. Infrared Spectroscopy

The purified powders of bisphthalocyanines were analyzed by FTIR using a Bruker Vertex 70 spectrometer, complemented with an attenuated total reflection (ATR) sampling accessory. The spectra were scanned in the 500–4000 cm^−1^ range with a resolution of 4 cm^−1^ at room temperature and under atmospheric pressure.

#### 3.3.2. Raman Spectroscopy

Raman spectra were recorded with a home-built Raman microscope at an excitation wavelength of 785 nm and using a 50× long working distance objective on a BX-51 microscope. Excitation was provided by an ONDAX LM-785 laser (75 mW at source), which was passed through a laser line clean-up filter (Semrock LL01-785), a $\frac{1}{2}$ λ retarder and a polarizing beam splitter to control power and then through a second $\frac{1}{2}$ λ retarder to control polarization. The spectra were the sum of 60 scans acquired with a typical acquisition of 0.5 to 1 s.

#### 3.3.3. Elemental Analysis

Elemental analysis was performed with a CHNS analyzer vario MICRO cube elemental. About 10 mg of each synthesized bisphthalocyanine was used.

#### 3.3.4. X ray Photoelectron Spectroscopy

X-ray photoelectron spectroscopy analysis was performed using a Surface Science SSX-100 ESCA instrument (Surface Science Instruments, Fisons, Ipswich, Suffolk, UK) with a monochromatic Al Kα X-ray source (hν = 1486.6 eV). The pressure in the measurement chamber was maintained at 1 × 10^−9^ mbar during data acquisition. The electron take-off angle with respect to the surface normal was 37° (and the resolution was set to 1.3 eV). The samples were dispersed in isopropanol by sonication for 10 min, and two small drops of the suspension were deposited on a homemade 150 nm thick gold film supported on mica [46] and left to dry in air. All XPS spectra were analyzed using a least squares curve-fitting program Winspec (developed at LISE laboratory of the University of Namur, Belgium). Spectral analysis included a Shirley background subtraction and fitting with peak profiles taken as a convolution of Gaussian and Lorentzian functions. All binding energies derived from deconvolution are reported ±0.1 eV.

#### 3.3.5. Ultraviolet–Visible Spectroscopy

Ultraviolet–visible spectra were measured in two aprotic polar solvents with high boiling points, 153 °C for N, N-dimethylformamide (99.8% purity) and 189 °C for dimethyl sulfoxide (99.9%) in the range of λ = 300–800 nm using a Cary 60 UV-vis spectrophotometer. Solutions were prepared with 1 mg of the product in 2 mL of the solvent. Since these were saturated solutions, a dilution was prepared from aliquots of 1 mL in 1 mL of each solvent.

#### 3.3.6. Electron Paramagnetic Resonance Spectroscopy

Electron paramagnetic resonance spectra were recorded at a scan on a Bruker EMXNano X-band spectrometer (Bruker, Billerica, MA, USA) with optical access to the cavity operating at 9.65 GHz with power 50 dB, gain 40 dB, sweep width 200 G, sweep time 60 s and modulation amplitude 1.0 G. The measurements were performed at room temperature.

#### 3.3.7. Thermal Analysis

Thermogravimetric analysis and differential thermal analysis curves were acquired by TA Instruments SDT 2960 (TA Instrumentsr, Etten-Leur, The Netherlands). Samples of approximately 5 mg were heated in an air flow of 100 mL·min^−1^ from (at room temperature) 10 °C min^−1^ to 1000 °C.

#### 3.3.8. Scanning Electron Microscopy

Scanning electron microscopy images were acquired on A FEI Nova NanoSEM 650 (Thermo Fisher Scientific, Eindhoven, The Netherlands) equipped with an Ametek EDAX-TLS EBSD system operating at 20 kV. The samples were analyzed in powder form, attached to carbon tape.

## 4. Conclusions

Unsubstituted yttrium, lanthanum, gadolinium, and terbium bisphthalocyanines were successfully synthesized by a solvothermal method presenting an alternative approach to conventional synthesis routes typically involving a phthalonitrile precursor. The compounds were synthesized in satisfactory yields of 68% for YPc_2_, 43% for LaPc_2_, 63% for GdPc_2_ and 62% for TbPc_2_. The structural integrity of the compounds as well as their purity were confirmed by FTIR, Raman and X-ray photoelectron spectroscopy as well as by elemental analysis. UV–visible spectroscopy corroborated their electronic properties. The EPR results revealed the presence of a dianionic and a monoanionic radial [M^3+^(Pc^2−^) (Pc^•−^)] ligand within the bisphthalocyanines. TGA and DTA indicated that the thermal decomposition starts around 350 °C. This new synthesis method opens the way to obtaining bisphthalocyanines without the need to use excessive amounts of solvents in their preparation and purification.

## Figures and Tables

**Table 1:** Yield and elemental analysis of rare earth double-decker phthalocyanine and amount of free-base phthalocyanine present in end product and elemental analysis.

MPc_2_ Complexes	Yield (%) ^a^	H_2_Pc (%) ^b^	Elemental Analysis (%)			
			C	H	N	M
YPc_2_	68.1	5.0	69.37 (69.00) ^c^	2.92 (2.89)	20.17 (20.12)	7.54 (7.98)
LaPc_2_	43.0	33.6	66.09 (66.04)	2.69 (2.65)	19.28 (19.25)	12.04 (11.93)
GdPc_2_	63.1	16.7	65.08 (65.04)	2.80 (2.73)	19.53 (18.96)	12.64 (13.3)
TbPc_2_	61.8	18.3	65.44 (64.92)	2.98 (2.72)	18.96 (18.93)	12.63 (13.4)

^a^ Based on Li_2_Pc (limiting reactant). ^b^ Calculated from the washed end product. ^c^ Theoretical percentage in parentheses.

## Data Availability

Data are contained within the article.

## Supplementary Materials

The following supporting information can be downloaded at https://www.mdpi.com/article/10.3390/molecules29112690/s1. Figure S1: Full FTIR spectra of free-base and rare earth double-decker phthalocyanines (YPc_2_, LaPc_2_, GdPc_2_ and TbPc_2_); Table S1: Characteristic IR bands (cm^−1^) of phthalocyanine for MPc_2_ (M = Y, La, Gd and Tb), as deduced from FTIR spectra recorded on powder samples; w = weak intensity; m = medium intensity; s = strong intensity; Table S2: Characteristic Raman bands (cm^−1^) of phthalocyanine for MPc_2_ (M = Y, La, Gd and Tb), as deduced from spectra recorded on powder samples with excitation at 633 nm; Table S3: The binding energies (BEs in eV) of the various contributions to the C 1s core-level region of yttrium, lanthanum, gadolinium and terbium bisphthalocyanine, Table S4: The binding energies (BEs in eV) of the various contributions to the N 1s and the metal 3d core-level regions of yttrium, lanthanum, gadolinium and terbium bisphthalocyanine; Table S5: The position of the B (Soret) and Q bands (λmax, nm) in the UV-vis spectra of double-decker phthalocyanines (MPc_2_ with M = Y, La, Gd and Tb) in DMF and DMSO.
