# Supplementary material for: Solvothermal Synthesis of Rare Earth Bisphthalocyanines
Source: Molecules. 2024 Jun 6;29(11):2690. doi: 10.3390/molecules29112690 (PMC11173967; doi:10.3390/molecules29112690)
Supplement: Supplementary file 1 [file molecules-29-02690-s001.zip › molecules-2998750-supplementary.pdf]

Supporting Information

Solvothermal Synthesis of Rare Earth Bisphthalocyanines

Lina M. Bolivar-Pineda [1,2,*], Carlos U. Mendoza-Domínguez [1], Petra Rudolf [2,*], Elena V. Basiuk [3] and Vladimir A. Basiuk [1,*]

[1] Instituto de Ciencias Nucleares, Universidad Nacional Autónoma de México, Circuito Exterior C.U., Ciudad de Mexico 04510, Mexico; cumd_2863@hotmail.com
[2] Zernike Institute for Advanced Materials, University of Groningen, Nijenborgh 4, 9747AG Groningen, The Netherlands
[3] Instituto de Ciencias Aplicadas y Tecnología, Universidad Nacional Autónoma de México, Circuito Exterior C.U., Ciudad de México 04510, Mexico; elena.golovataya@icat.unam.mx
* Correspondence: linabolivar12@gmail.com (L.M.B.-P.); p.rudolf@rug.nl (P.R.); basiuk@nucleares.unam.mx (V.A.B.)

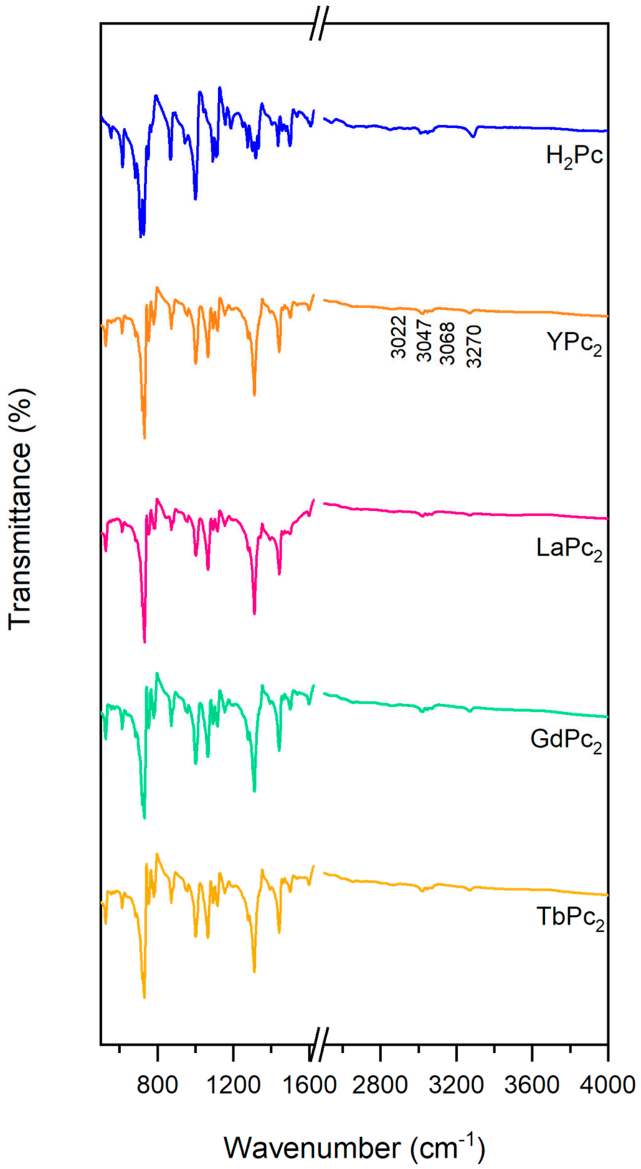

Figure S1. Full FTIR spectra of free-base and rare earth double-decker phthalocyanines (YPc_2, $LaPc_2$, $GdPc_2$ and $TbPc_2$).

Table S1. Characteristic IR bands (cm^{-1}) of phthalocyanine for MPc_2 (M = Y, La, Gd and Tb) as deduced from FTIR spectra recorded on powder samples; w=weak intensity; m=medium intensity; s=strong intensity.

**YPc_2**	**$LaPc_2$**	**$GdPc_2$**	**$TbPc_2$**	**Assignment** [1–4]
526w	526w	526w	527w	Benzene radial
613 w	613w	613w	613w	Pc breathing
685 w	685w	685w	685w	Pc breathing
719m	719m	719m	719m	C-H out of plane bending
731s	731s	731s	731s	C-H out of plane bending
754w	754w	754w	754w	Pc ring
781w	787w	781w	781w	Pc ring
874w	874w	874w	874w	Coupling of isoindole deformation and aza stretching
957w	957w	957w	957w	Benzene
1001m	1003m	1001m	1001m	Pyrrole-N in-plane bending
1066m	1066m	1065m	1065m	Coupling of isoindole deformationand aza stretching
1093w	1093w	1094w	1093w	C-H in plane bending
1117w	1117w	1117w	1117w	Isoindole breathing
1155w	1155w	1155w	1155w	C-H in plane bending
1279w	1279w	1279w	1279w	C-H in plane bending
1311s	1311s	1311s	1311s	Pyrrole stretching
1394w	1392w	1394w	1394w	Isoindole stretching
1443m	1443m	1443m	1443m	Isoindole stretching
1464w	1464w	1464w	1464w	Isoindole stretching
1477	1477w	1479w	1477w	Isoindole stretching
1500w	1498w	1500w	1500w	Coupling of pyrrole and aza stretching
1537w	----	1537w	1537w	Benzene stretching
1599w	1599w	1599w	1599w	Benzene stretching
3022	3022w	3022w	3022w	C-H stretching aromantic)
3047	3047w	3047w	3045w	C-H stretching (aromatic)
3068	3068w	3068w	3068w	C-H stretching (aromatic)
3270	3269w	3270w	3271w	C-H stretching (aromatic)

Table S2. Characteristic Raman bands (cm^{-1}) of phthalocyanine for MPc_2 (M = Y, La, Gd and Tb) as deduced from spectra recorded on powder samples with excitation at 633 nm

**YPc_2**	**$LaPc_2$**	**$GdPc_2$**	**$TbPc_2$**	**Assignment** [2,5–8]
476	480	480	476	Pc breathing
----	----	----	541	Pc breathing
572	576	576	572	Pc breathing
684	688	689	683	Pc breathing
721	725	724	721	Aza Isoindole stretching
736	739	740	736	C-H wag
795	798		793	C=N aza stretching
807	811	811	806	C=N aza stretching
952	958	958	954	C-H in plane bending
969	997	----	----	C-H in plane bending
1007	1011	1013	1005	CH in plane bending, isoindole stretching
1035	1038	1039	1033	C-H in plane bending
1101	1104	1106	1100	C-H in plane bending
1142	1145	1145	1141	Pyrrole breathing
1161	1165	1166	1160	C-H in plane bending
1182	----	1183	1180	Isoindole deformation
1219	1222	1222	1219	C-H in plane bending
1258	1266	1262	1259	Isoindole stretching and deformation, aza stectching
1295	1296	1299	1293	C-H in plane bending, isondole deformation
1312	1316	1316	1311	Isoindole stretching
1340	1344	1344	1339	Aza stretching, isoindole deformation
1370	1373	1374	1368	Isoindole stretching and deformation, aza stretching
1422	1424	1425	1420	Isoindole stretching
1449	1451	1453	1448	Isoindole stretching
1475	1478	1477	1474	Isoindole stretching
1540	1544	1536	1539	Aza and pyrrole stretching

Table S3. Binding energies (BE in eV) of the various contributions to the C 1*s* core level region of yttrium, lanthanum, gadolinium, and terbium bisphthalocyanine

		C 1*s* (eV)		
**Sample**	**C=C**	**N-C=N**	**Shake-up$_{C=C}$**	**Shake-up$_{N-C=N}$**
YPc_2	284.7	285.8	287.2	289.0
$LaPc_2$	284.6	285.8	287.5	289.1
$GdPc_2$	284.5	285.6	287.3	289.1
$TbPc_2$	284.7	285.9	287.5	289.4

Table S4. Binding energies (BE in eV) of the various contributions to the N 1*s* and the metal 3*d* core level regions of yttrium, lanthanum, gadolinium, and terbium bisphthalocyanine

		**N 1s (eV)**		**M 3$d_{5/2}$ (eV)**
**Sample**	**M-N**	**C=N-C**	**Shake-up**	
YPc_2	398.2	399.9	401.6	157.3
$LaPc_2$	398.1	399.6	401.2	835.8
$GdPc_2$	398.1	399.6	401.3	1186.5
$TbPc_2$	398.0	399.5	401.0	1242.9

Table S5. Position of the B (Soret) and Q bands (λmax, nm) in the UV-vis spectra of double-decker phthalocyanines (MPc_2 with M = Y, La, Gd and Tb) in DMF and DMSO.

**Bisphthalocyanine**	**Solvent**	**Absorption (nm)**			
		**Q band**		**B-band**	**Radical**
**YPc_2**	DMF	665	603	333	--
	DMSO	665	--	333	448
**$LaPc_2$**	DMF	662	599	328	--
	DMSO	665	602	328	448
**$GdPc_2$**	DMF	662	599	329	--
	DMSO	667	602	328	446
**$TbPc_2$**	DMF	662	599	328	--
	DMSO	665	601	326	--

References

1. Souto, J.; Tomilova^, L.; Aroca, R.; Desaja, J.A. Spectroscopic Studies of Langmuir-Blodgett Monolayers of Praseodymium Bisphthalocyanines. *Langmuir* **1992**, *8*, 942–946.

2. Lu, F.; Bao, M.; Ma, C.; Zhang, X.; Arnold, D.P.; Jiang, J. Infrared Spectra of Phthalocyanine and Naphthalocyanine in Sandwich-Type (Na)Phthalocyaninato and Porphyrinato Rare Earth Complexes. Part 3. The Effects of Substituents and Molecular Symmetry on the Infrared Characteristics of Phthalocyanine in Bis(Phthal. *Spectrochim. Acta Part A Mol. Biomol. Spectrosc.* **2003**, *59*, 3273–3286, doi:10.1016/S1386-1425(03)00158-6.

3. Jiang, J.; Arnold, D.P.; Yu, H. Infra-Red Spectra of Phthalocyanine and Naphthalocyanine in Sandwich-Type (Na)Phthalocyaninato and Porphyrinato Rare Earth Complexes. *Polyhedron* **1999**, *18*, 2129–2139.

4. Kratochvílová, I.; Šebera, J.; Paruzel, B.; Pfleger, J.; Toman, P.; Marešová, E.; Havlová; Hubík, P.; Buryi, M.; Vrňata, M.; et al. Electronic Functionality of Gd-Bisphthalocyanine: Charge Carrier Concentration, Charge Mobility, and Influence of Local Magnetic Field. *Synth. Met.* **2018**, *236*, 68–78, doi:10.1016/J.SYNTHMET.2018.01.007.

5. Jiang, J.; Bao, M.; Rintoul, L.; Arnold, D.P. Vibrational Spectroscopy of Phthalocyanine and Naphthalocyanine in Sandwich-Type (Na)Phthalocyaninato and Porphyrinato Rare Earth Complexes. *Coord. Chem. Rev.* **2006**, *250*, 424–448, doi:10.1016/J.CCR.2005.09.009.

6. Suzuki, A.; Oku, T. Effects of Central Metal on Electronic Structure, Magnetic Properties, Infrared and Raman Spectra of Double-Decker Phthalocyanine. *Appl. Surf. Sci.* **2016**, *380*, 127–134, doi:10.1016/j.apsusc.2016.02.026.

7. Zheng, W.; Wang, B.B.; Lai, J.C.; Wan, C.Z.; Lu, X.R.; Li, C.H.; You, X.Z. Electrochromic Properties of Novel Octa-Pinene Substituted Double-Decker Ln(III) (Ln = Eu, Er, Lu) Phthalocyanines with Distinctive near-IR Absorption. *J. Mater. Chem. C* **2015**, *3*, 3072–3080, doi:10.1039/C5TC00020C.

8. Zhang, Y.; Cai, X.; Zhou, Y.; Zhang, X.; Xu, H.; Liu, Z.; Li, X.; Jiang, J. Structures and Spectroscopic Properties of Bis(Phthalocyaninato) Yttrium and Lanthanum Complexes: Theoretical Study Based on Density Functional Theory Calculations. *J. Phys. Chem. A* **2007**, *111*, 392–400, doi:10.1021/jp066157g.
